# Pituitary Adenoma: *SSTR2* rs2236750, *SSTR5* rs34037914, and *AIP* rs267606574 Genetic Variants, Serum Levels, and Ki-67 Labeling Index Associations

**DOI:** 10.3390/medicina60081252

**Published:** 2024-08-01

**Authors:** Greta Gedvilaite-Vaicechauskiene, Loresa Kriauciuniene, Arimantas Tamasauskas, Vita Rovite, Ilona Mandrika, Sheng-Nan Wu, Chin-Wei Huang, Lina Poskiene, Rasa Liutkeviciene

**Affiliations:** 1 Neuroscience Institute, Medical Academy, Lithuanian University of Health Sciences, Eiveniu 2, 50161 Kaunas, Lithuania; greta.gedvilaite@lsmuni.lt (G.G.-V.); loresa.kriauciuniene@lsmuni.lt (L.K.); arimantas.tamasauskas@lsmuni.lt (A.T.); 2 Latvian Biomedical Research and Study Centre (BMC), LV-1067 Rīga, Latvia; vita.rovite@biomed.lu.lv (V.R.); ilona@biomed.lu.lv (I.M.); 3 Department of Neurology, National Cheng Kung University Hospital, Tainan City 704, Taiwan; snwu@mail.ncku.edu.tw (S.-N.W.); huangcw@mail.ncku.edu.tw (C.-W.H.); 4 Department of Pathology, Medical Academy, Lithuanian University of Health Sciences, 50161 Kaunas, Lithuania; lina.poskiene@lsmuni.lt

**Keywords:** pituitary adenoma, Ki-67 labeling index, *SSTR2* rs2236750, *SSTR5* rs34037914, *AIP* rs267606574 polymorphisms, serum levels

## Abstract

*Background and Objectives:* This study explores the complex pathogenesis of pituitary adenomas (PAs), prevalent intracranial tumors in the pituitary gland. Despite their generally benign nature, PAs exhibit a diverse clinical spectrum involving hormone hypersecretion and varying invasiveness, hinting at multifaceted molecular mechanisms and abnormalities in tumorigenesis and gene regulation. *Materials and Methods:* The investigation focuses on the Ki-67 labeling index, *SSTR2* rs2236750, *SSTR5* rs34037914, and *AIP* rs267606574 polymorphisms, alongside serum levels of SSTR2, SSTR5, and AIP, to discern their association with PAs. The Ki-67 labeling index was assessed using immunohistochemical analysis with the monoclonal antibody clone SP6, representing the percentage of tumor cells showing positive staining. Genotyping was performed via real-time polymerase chain reaction, and serum levels were analyzed using ELISA. The study included 128 PA patients and 272 reference group subjects. *Results:* The results derived from binary logistic regression analysis revealed an intriguing correlation between the *SSTR2* rs2236750 AG genotype and approximately a 1.6-fold increased likelihood of PA occurrence. When analyzing *SSTR5* rs34037914, statistically significant differences were found between Micro-PA and the reference group (*p* = 0.022). Additionally, the *SSTR5* rs34037914 TT genotype, compared with CC + CT, under the most robust genetic model (selected based on the lowest AIC value), was associated with a 12-fold increased odds of Micro-PA occurrence. However, it is noteworthy that after applying Bonferroni correction, these findings did not retain statistical significance. *Conclusions:* Consequently, while this study hinted at a potential link between *SSTR2* rs2236750 and pituitary adenoma development, as well as a potential link between *SSTR5* rs34037914 and Micro-PA development, it underscored the need for further analysis involving a larger cohort to robustly validate these findings.

## 1. Introduction

Pituitary adenomas (PAs) develop mainly in the anterior lobe of the pituitary gland and are usually slow-growing, benign tumors [1]. While specific data on the prevalence of PAs in the Lithuanian population are not available, global studies indicate an estimated prevalence of approximately 1 in 1000 individuals. However, the frequency and prevalence of pituitary adenomas can vary by region [2]. PAs are classified according to their size (microadenomas, macroadenomas, and giant tumors) and their functionality or anatomical extent [3]. Microadenomas are smaller than 10 mm, macroadenomas are larger than 10 mm, and giant tumors are larger than 40 mm [1]. Distinguishing between microadenomas and macroadenomas is clinically significant, as it directly influences management strategies and prognostic considerations. Microadenomas, due to their smaller size, often present with less invasive behavior and are frequently managed with less aggressive treatment approaches, such as pharmacotherapy or regular monitoring. In contrast, macroadenomas tend to exhibit more invasive characteristics, including potential encroachment on surrounding structures and more complex surgical challenges [1,3]. Clinically, PAs are categorized into non-functional pituitary adenomas (NFPA) and functional pituitary adenomas (FPA), with functional adenomas accounting for about 60–70% of cases and nonfunctional adenomas making up the remaining 30–40% [4].

Compared to FPAs, NFPAs are more aggressive and more challenging to recognize, as they are asymptomatic and only appear when the adenomas enlarge and begin to press on the surrounding structures [5]. Functional PAs increase the secretion of certain adenohypophyseal hormones (e.g., growth hormone, thyroid-stimulating hormone, adrenocorticotropic hormone, follicle-stimulating hormone, and luteinizing hormone) [6,7]. One of the most important behaviors of PA is its invasiveness, which manifests itself in the destruction of surrounding structures and triggers numerous complications. It has also been shown that invasiveness can indicate poor prognosis [8].

Around 5% of pituitary tumors are hereditary, with genetic mutations being a rare cause. The medical community recognizes the recurrence of pituitary adenomas, although there are few comprehensive studies on the recurrence rate and associated clinical factors [1]. Tumor prognosis is highly dependent on the number of mitoses. In cases where few mitoses are detectable in aggressive cases, Ki-67 represents an alternative essential factor in assessing tumor proliferation. According to the latest World Health Organization classification, the Ki-67 labeling index is an important prognostic indicator for pituitary adenomas [9]. Since the 1980s, the relationship between Ki-67 and tumor size and type, invasiveness, recurrence, and malignancy has been studied, often with conflicting results, except for a consistently positive association with tumor invasiveness [10,11].

The pathogenesis of PA is multifactorial: many factors are involved in developing this disease, including external environmental factors, pathological changes in PA that are necessary for its development, and genetic factors. Somatostatin receptors (SSTRs) exert their effects through various pathways, including inhibition of adenylyl cyclase, stimulation of rectifying K^+^ channels, reduction of Ca^2+^ channel conductance, and enhancement of tyrosine phosphatase activity [12]. These SSTR subtypes are widely expressed in both rodent and human tissues, with expression observed in the central nervous system and hypothalamus [13]. In the context of neuroendocrine tumors (NETs), somatostatin receptor subtype 5 (SSTR5) plays a crucial role, but understanding its regulatory mechanisms is still incomplete. A study by Pedraza-Arevalo et al. provided new insights and suggested that SSTR5 expression in pituitary NETs may be epigenetically regulated by the antisense transcript SSTR5-AS1 and DNA methylation [14]. Recently, scientists reported a remarkable finding regarding the membrane expression of somatostatin receptor subtype 2 (SSTR2) in neuroendocrine tumor cells, which is increased approximately 20-fold compared to normal cells. These convincing results underline that SSTR2 is significantly and widely present in neuroendocrine tumors [15]. Consequently, these results present SSTR2 as a promising target for developing innovative therapeutic strategies for treating neuroendocrine tumors [15].

Mutations of the aryl-hydrocarbon receptor-interacting protein (AIP) have been extensively investigated, particularly in individuals predisposed to familial and sporadic pituitary PitNETs [16]. Functioning as a suppressor gene, AIP encodes a 330 amino-acid-long protein involved in the cAMP phosphodiesterase signaling pathway [17]. The most prevalent AIP variants (AIPvar) encompass nonsense and missense mutations, deletions, insertions, and mutations at the splice site and promoter, as well as large deletions [18]. These variations often result in a truncated protein or, less frequently, impact the tetratricopeptide repeat (TPR) domains or the C-terminal α-helix [19]. In addition, loss of heterozygosity (LOH) in tumor tissue at the site of the AIP gene in the 11q13 region has been observed in patients with germline AIPvar [17]. Some AIPvar are rare alterations with no pathogenic effects and no impact on protein function. The distinction between these two aspects is important because rare genetic alterations can also be found in healthy controls [18]. Although few germline mutations are known to contribute to the inherited risk of pituitary tumors, recent findings highlight the importance of *AIP* gene mutations in familial cases and their potential role in sporadic somatotropinomas. Furthermore, the genetic associations with acromegaly and SSTRs have only been explored to a limited extent [20].

Therefore, this study aims to investigate the Ki-67 labeling index, *SSTR2* rs2236750, *SSTR5* rs34037914, and *AIP* rs267606574 polymorphisms, serum SSTR2, SSTR5, and AIP levels, and their association with the development of pituitary adenomas.

## 2. Materials and Methods

The study was conducted in the Laboratory of Ophthalmology, Lithuanian University of Health Sciences. Kaunas Regional Biomedical Research Ethics Committee approved the study (approval number: BE-2-47, dated 25 December 2016). All participants were introduced to the structure and objectives of the present study before the execution. An informed consent form was obtained from all subjects involved in the study.

The *SSTR2* rs2236750 variant is located in a non-coding region, while the *SSTR5* rs34037914 variant is a synonymous variant. We acknowledge that these variants may not directly influence protein structure or function. However, previous studies have suggested that variants in non-coding regions or synonymous variants could still affect gene expression, splicing patterns, or mRNA stability, thereby potentially impacting disease susceptibility or progression. Regarding the *AIP* rs267606574 variant, we acknowledge that it represents an in-frame deletion of three nucleotides and is not commonly found in population databases. We included this variant in our study based on preliminary evidence suggesting its potential association with PA susceptibility or clinical features. However, all genotyped individuals in our study exhibited the normal sequence.

### 2.1. DNA Extraction and Genotyping

The DNA was extracted from peripheral venous blood samples (leucocytes) collected in 200 µL test tubes utilizing the silica-based membrane technology using a genomic DNA extraction kit (GeneJET Genomic DNA Purification Kit, Thermo Fisher Scientific, Vilnius, Lithuania), based on the manufacturer’s recommendations. 

Single-nucleotide polymorphisms of *SSTR2* rs2236750, *SSTR5* rs34037914, and *AIP* rs267606574 were carried out using the real-time polymerase chain reaction (RT-PCR) method. TaqMan^®^ Genotyping assays were used to determine SNPs according to the manufacturer’s protocols by a StepOne Plus (Applied Biosystems, Waltham, MA, USA). A 5% subset of samples underwent repetitive analysis for all three SNPs to ensure accuracy, confirming consistent genotyping results between the initial and repetitive assessments.

Primers and Probes:

- *SSTR2* rs2236750: TaqMan^®^ Genotyping Assay (Assay ID: C__15954985_10; Catalog # 4351379), with specific primers and fluorescently labeled probes designed for this SNP.
- *SSTR5* rs34037914: TaqMan^®^ Genotyping Assay (Assay ID: C__26059313_10; Catalog # 4351379), with specific primers and fluorescently labeled probes designed for this SNP.
- *AIP* rs267606574: TaqMan^®^ Genotyping Assay (Assay ID: C_189786883_20; Catalog # 4351379), with specific primers and fluorescently labeled probes designed for this SNP.

### 2.2. Serum Levels’ Measurement

Serum levels of Human Somatostatin Receptor 2 (SSTR2) with capture antibody specific to SSTR2, Human Somatostatin Receptor 5 (SSTR5) with capture antibody specific to SSTR5, and Human Aryl Hydrocarbon Receptor-Interacting Protein (AIP) with capture antibody specific to AIP were measured using enzyme-linked immunosorbent assay (ELISA) kits, employing a sandwich assay methodology. Each ELISA kit consisted of a 96-well plate pre-coated with specific antibodies tailored to capture the target proteins. The procedure followed a standardized protocol, as follows:

- Sample incubation. Test samples, along with standards of known concentrations and biotin-conjugated reagents, were added to the wells and allowed to incubate. This step facilitated the binding of the target proteins to the immobilized capture antibodies.
- Enzymatic reaction initiation. Subsequent addition of horseradish peroxidase (HRP)-conjugated reagents initiated an enzymatic reaction. This reaction generated a measurable signal directly proportional to the concentration of the target protein bound to the antibodies.
- Colorimetric detection. The enzymatic reaction catalyzed by HRP resulted in the conversion of a colorless substrate, tetramethylbenzidine (TMB), to a blue-colored product. Upon addition of an acidic stop solution, the reaction was terminated, causing the color to change to yellow. The intensity of this yellow color was directly correlated with the concentration of the target protein in the sample.
- Optical density measurement. The absorbance of the resulting yellow color was measured at 450 nm using a microplate reader. This optical density (OD) value served as the basis for precise concentration calculations, particularly within blood serum samples.

The process involved a standardized method of measurement and calculation. Reference standard readings were utilized to generate a standard curve for each respective protein (SSTR2, SSTR5, and AIP). By comparing the OD values of the samples to the standard curve, concentrations of the target proteins in the serum samples were interpolated accurately. This approach ensured robust and reproducible quantification of serum levels of SSTR2, SSTR5, and AIP, providing valuable insights into their potential biomarker roles in the context of our study.

### 2.3. Ki-67

The Ki-67 labeling index (LI) was determined through immunohistochemical analysis using the monoclonal antibody (clone SP6; Spring Bioscience Corporation, Pleasanton, CA, USA). This index represents the percentage of tumor cells showing positive staining.

The evaluation of Ki-67 LI took place at the Clinic of Pathological Anatomy at LUHS and was conducted by a qualified pathologist. The analysis of protein biomarkers followed the immunohistochemical analysis protocol on paraffin sections, utilizing the Ventana BenchMark XT staining procedure from Ventana Medical Systems in Tucson, AZ, USA. Paraffin sections underwent dewaxing with Ventana reagent, followed by antigenic epitope restoration using Ventana Cell Conditioning Solution (pH 8.4) at 100 °C for 60 min. Monoclonal antibodies were applied to the sections for 32 min at 37 °C and detected using the Ventana iVIEW DAB Detection Kit. The immunohistochemical reaction concluded with contrasting sections using Gill’s Hematoxylin Solution, coloration with a bluing reagent of an aqueous solution of buffered lithium carbonate, and covering with glass slides.

### 2.4. Study Group

**Figure 1:**
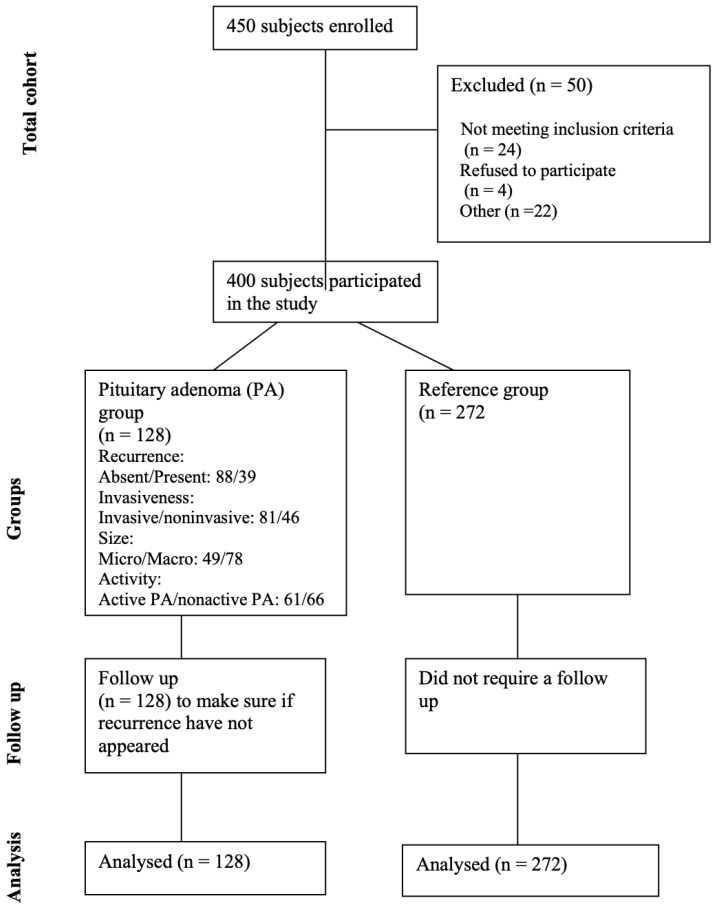
Flow diagram for study participants.

The study comprised 400 subjects categorized into a reference group (n = 272) and a group of patients with pituitary adenoma (n = 128). The reference group was adjusted concerning gender and age to match the demographics of the pituitary adenoma group (with *p*-values of 0.077 and 0.821, respectively). Demographic information for all study subjects is detailed in Table 1, providing a comprehensive overview of the characteristics within both groups. Figure 1 shows the study flow diagram for the study participants.

Patients with PAs were selected based on the following inclusion criteria:

1. Confirmed diagnosis: only patients with a confirmed diagnosis of PA through imaging (MRI/CT) and/or histopathological examination were included.
2. Age and health status: patients were included regardless of age (age of 18 years or older) and overall good health status, provided they met the diagnostic criteria for PA.
3. Our study exclusively included patients whose PA was investigated and diagnosed based on clinical symptoms.
4. Overall good health condition of the patient.
5. Absence of other brain tumors, tumors in other locations, intracranial infections, demyelinating lesions, or cerebrovascular diseases.
6. Consent: an informed consent form was obtained from all patients included in the study.

For bias elimination, we employed several strategies to minimize bias in our study, as follows:

1. Comprehensive data collection: we included a broad range of patients with different types of PAs (functional and non-functional) to ensure diversity and representativeness.
2. Standardized diagnostic criteria: Consistent diagnostic criteria were applied to all patients to ensure uniformity in inclusion. PAs in our study were detected while investigating symptomatic patients. The diagnosis was based on clinical symptoms suggestive of PAs, such as hormonal imbalances, visual disturbances, or headaches.

Patients with PAs exclusion criteria were as follows:

1. Non-confirmed diagnosis: patients without a confirmed diagnosis of pituitary adenoma (PA) through imaging (MRI/CT) and/or histopathological examination were excluded.
2. Age and health status: patients younger than 18 years of age, or those with significant health issues that could impact their participation or the study results, were excluded.
3. Non-clinical diagnosis: patients whose PAs were not investigated and diagnosed based on clinical symptoms were excluded.
4. Poor health condition: patients in poor overall health, as determined by their clinical assessment, were excluded.
5. Other brain or systemic conditions: patients with other brain tumors, tumors in other locations, intracranial infections, demyelinating lesions, or cerebrovascular diseases were excluded.
6. Lack of consent: patients who did not provide informed consent were excluded from the study.

Reference group inclusion criteria were as follows:

1. Healthy status: individuals must be in overall good health, with no history of pituitary adenomas or other significant health issues that could affect the study’s results.
2. Age: individuals must be 18 years of age or older to ensure comparability with the patient group.
3. No brain or systemic disorders: individuals must have no history of brain tumors, tumors in other locations, intracranial infections, demyelinating lesions, cerebrovascular diseases, or any other major systemic disorders.
4. No prior diagnosis of pituitary disorders: individuals must not have a history or diagnosis of pituitary disorders, including pituitary adenomas, as confirmed through imaging or clinical evaluation.
5. Consent: informed consent must be obtained from all individuals included in the reference group.

Reference group exclusion criteria included:

1. Presence of health issues: individuals with significant health conditions, including pituitary disorders, brain tumors, or major systemic diseases, were excluded.
2. Age restrictions: individuals younger than 18 years were excluded to match the age range of the patient group.
3. History of pituitary or brain disorders: individuals with a history of pituitary disorders, brain tumors, or other relevant conditions were excluded.
4. Informed consent: individuals who did not provide informed consent were excluded from the reference group.

Within the PA group, further subdivisions were performed based on several key factors, including recurrence, invasiveness, size, and hormonal activity. Histopathological examination allowed for the classification into invasive and noninvasive subgroups, while analysis of hormone levels in the blood serum led to the creation of active and nonactive PA groups. Recurrence was identified if there was observed enlargement of a residual tumor, or the appearance of new growth documented in follow-up MRIs over a period of five years.

Moreover, PAs were categorized based on size, with those measuring 10 mm or larger classified as macroadenomas, and those smaller than 10 mm termed microadenomas. This thorough categorization process ensured precise delineation within the PA group for comprehensive analysis and understanding.

### 2.5. Statistical Analysis

The demographic characteristics between the reference and pituitary adenoma (PA) groups underwent comparison using a range of statistical tests, including the Pearson Chi-square test, Student’s *t*-test, and Mann–Whitney U test. The frequencies of genotypes and alleles for *SSTR2* rs2236750, *SSTR5* rs34037914, and *AIP* rs267606574 were presented as percentages. Binary logistic regression analysis assessed the association of selected SNPs with PA occurrence, estimating odds ratios (OR) and 95% confidence intervals (CI). Various inheritance models (co-dominant, dominant, recessive, over-dominant, and additive genetic models) were assessed, with the model having the lowest Akaike information criterion (AIC) chosen as the most appropriate. The most robust genetic models were identified based on the lowest AIC value. Nonparametric Mann–Whitney U tests were used for comparisons when data distribution deviated from normality. 

The Bonferroni adjustment was used to modify the significance level for multiple comparisons (*p* = 0.025 (0.05/2)). All statistical analysis was performed using SPSS version 29.0 software (Statistical Package for the Social Sciences for Windows, Inc., Chicago, IL, USA).

### 2.6. Strengths and Limitations of the Study

The strength of the present study was the inclusion of a large study population (a total of 400 subjects, including 128 patients with PA, as PA is considered a rare disease in the Lithuanian population (ORPHA:91349), and 272 healthy controls as a reference group), age and gender matching of the patient and control groups, and including Ki-67 analysis. These features of the study ensured a comprehensive analysis of the associations between the selected gene polymorphisms *SSTR2* rs2236750, *SSTR5* rs34037914, and *AIP* rs267606574 and the SSTR2, SSTR5, and AIP serum levels they produce. Most of the studies presented in the literature provided immunohistochemistry results but not gene analysis and analysis of serum levels, so SNP and serum levels’ analysis is worthy and important for understanding the pathogenesis of PA.

Several limitations must be considered in the present study. The sample size for analyzing serum concentrations of SSTR2, SSTR5, and AIP was rather limited and too small (40 patients with PA and 40 healthy controls) to achieve the desired significance. Further studies with a sufficiently large sample size are recommended to confirm the possible role of serum concentrations of SSTR2, SSTR5, and AIP in the development of PA. Of course, we also need to consider all subtypes of PA, such as the invasiveness of PA, recurrences, micro/macroadenomas, and hormones. Immunohistochemistry of SSTR2, SSTR5, and AIP must also be performed. However, this is planned as a specific task for our future investigations.

## 3. Results

The frequencies of genotypes and alleles for the following single-nucleotide polymorphisms (SNPs): *SSTR2* rs2236750, *SSTR5* rs34037914, and *AIP* rs267606574, were analyzed within the study groups. However, there were no statistically significant differences in the distribution of genotypes and alleles between patients with PA and the reference group for the selected SNPs (Table 2).

The Hardy–Weinberg equilibrium (HWE) test results demonstrated that genotypes of *SSTR2* rs2236750 and *SSTR5* rs34037914 in the reference group did not deviate from HWE (*p* > 0.05). However, we identified that *AIP* rs267606574 has only one genotype, which is not in HWE (Table 3). Regarding these findings, we excluded this SNP from the following analysis.

Binary logistic regression analysis revealed that *SSTR2* rs2236750 AG genotype vs. AA and AG vs. AA + GG was associated with about a 1.6-fold increased odds of PA occurrence under the co-dominant and over-dominant genetic models (OR = 1.602; CI: 1.015–2.527; *p* = 0.043; OR = 1.550; CI: 1.013–2.373; *p* = 0.044, respectively). However, after Bonferroni correction, these findings did not remain statistically significant (Table 4).

The frequencies of genotypes and alleles for the selected SNPs were analyzed within the study groups, stratified by gender; however, no statistically significant results were found neither in females (Table 5) nor in males (Table 6).

Binary logistic regression analysis was conducted in patients with PA and the reference group to investigate the associations of selected SNPs with PA occurrence by gender. The analysis did not reveal any statistically significant results when analyzing gender (Table 7) and (Table 8).

*SSTR2* rs2236750 and *SSTR5* rs34037914 genes’ single-nucleotide polymorphisms were analyzed to evaluate the associations with pituitary adenoma size. Only *SSTR5* rs34037914 showed statistically significant results between the groups: we found statistically significant differences between Micro-PA compared with the reference group (CC, CT, and TT: 87.5%, 12.1%, and 0.4% vs. 88.9%, 6.4%, and 4.3%; *p* = 0.022; Table 9).

Also, binary logistic regression analysis was conducted for Micro-PA and Macro-PA, as well as for the reference group. The results revealed the following associations: the *SSTR5* rs34037914 TT genotype, compared with CC + CT, under the most robust genetic model (selected based on the lowest AIC value), was associated with a 12-fold increased odds of Micro-PA occurrence (OR 12.044; 95% CI: 1.070–135.599; *p* = 0.044). However, these results did not remain statistically significant after Bonferroni correction was applied (Table 10).

The study evaluated serum levels of SSTR2, SSTR5, and AIP in patients with PA compared to a reference group. However, no statistically significant differences were found between the PA patients and the reference group for any of these markers.

**Figure 2:**
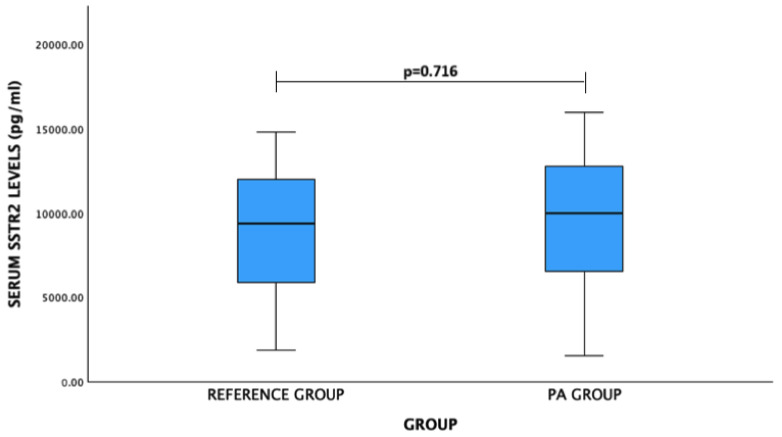
Serum SSTR2 levels (pg/mL) in PA and reference groups. Student’s *t* test was used.

For SSTR2 levels: PA patients vs. reference group: mean (standard deviation)—9401.21 (4279.74) pg/mL vs. 9078.18 (3627.34) pg/mL, *p* = 0.716. No significant difference was observed (Figure 2).

**Figure 3:**
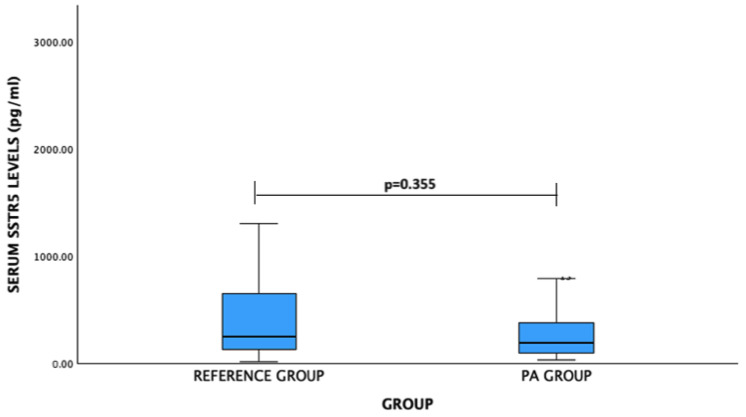
Serum SSTR5 levels (pg/mL) in PA and reference groups. Mann–Whitney U test was used.

For SSTR5 levels: PA patients vs. reference group: median (IQR)—194.15 (297.39) pg/mL vs. 250.94 (537.97) pg/mL, *p* = 0.355. No significant difference was observed (Figure 3).

**Figure 4:**
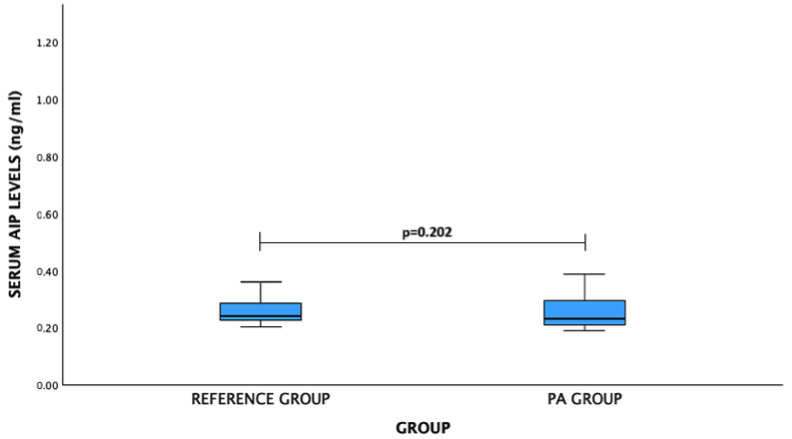
Serum AIP levels (ng/mL) in PA and reference groups. Mann–Whitney U test was used.

For AIP levels: PA patients vs. reference group: median (IQR)—0.232 (0.089) ng/mL vs. 0.241 (0.059) ng/mL, *p* = 0.202. No significant difference was observed (Figure 4).

Additionally, when considering gender differences, SSTR2 levels did not differ significantly between PA and reference group females or males (Table 11). SSTR5 levels did not differ significantly between PA and reference group females or males (Table 12). Also, AIP levels did not differ significantly between PA and reference group females or males (Table 13).

### Ki-67 Labeling Index

Here, 79 PA tissue samples were analyzed. The Ki-67 LI was evaluated in 41 women (51.9%) and 38 men (48.1%). The results showed that there was no significant difference in the Ki-67 LI between women and men (*p* = 0.301).

Immunohistochemistry for Ki-67 revealed an LI < 1% in 67.1% of patients with PA, a Ki-67 LI 1% in 13.9%, and a Ki-67 LI > 1% in 19.0% of patients. Further analyses revealed no statistical significance with regard to tumor invasiveness (*p* = 0.717; Table 14), recurrence (*p* = 0.843; Table 15), activity (*p* = 0.378; Table 16), or size (*p* = 0.492; Table 17). The analysis of the Ki-67 LI with the indicated genetic variations (*SSTR2* rs2236750, *SSTR5* rs34037914, and *AIP* rs267606574) also revealed no statistically significant correlations, as indicated in Table 18.

## 4. Discussion

In this research, we analyzed the Ki-67 labeling index (LI), *SSTR2* rs2236750, *SSTR5* rs34037914, and *AIP* rs267606574 polymorphisms, as well as serum SSTR2, SSTR5, and AIP levels, in association with PAs. 

The Ki-67 LI is considered an essential marker for categorizing pituitary neuroendocrine tumors (PitNETs) into different prognostic groups, although its definitive prognostic value in PitNETs remains under investigation [21,22]. The role of Ki-67 as a prognostic marker has been studied extensively, with some reports suggesting its integration with morphological and radiological evidence of local infiltration for better prognostication. Typical Ki-67 indices in PAs range from 1% to 2%, with values above 3% being rare [23,24]. Petry’s study reported that Ki-67 ranged from 0 to 30% [25], similar to the values described by Salehi et al. (less than 1% to 23%) [21] and Padrão (between 0 and 36.9%) [26]. Additionally, other studies reported Ki-67 values of 36% (Padrão et al.) [26], 29% (Petry et al.) [25], and 12% (Magagna-Poveda et al.) [27]. 

In our study, we analyzed 79 PA tissue samples. Immunohistochemistry for Ki-67 revealed an LI < 1% in 67.1% of patients with PA, a Ki-67 LI of 1% in 13.9%, and a Ki-67 LI > 1% in 19.0% of patients. However, further analyses revealed no statistical significance concerning tumor invasiveness, recurrence, activity, or size. Additionally, no statistically significant correlations were found between Ki-67 LI and the genetic variations (*SSTR2* rs2236750, *SSTR5* rs34037914, and *AIP* rs267606574). Similarly, tumor size [28] and the presence or absence of hormone hypersecretion [29] did not differ in Ki-67 LI expression in Sánchez-Tejada and colleagues’ study. 

Regarding genetic analysis, SSTR are G-protein-coupled receptors encoded by five different genes (*SSTR1–5*), and all five genes are expressed in normal adult human pituitary glands [30]. Neto and colleagues described that in PAs, SSTR expression is highly variable within and between tumor subtypes [31]. Wildemberg et al. demonstrated that in clinically non-functioning pituitary adenomas (NFPA), the SSTR2 mRNA transcripts were expressed in the majority of tumors, while the SSTR5 mRNA transcripts were expressed in a subset of tumors [32]. Two other researchers’ groups found that in somatotropinomas and normal pituitary glands, the mRNA transcripts and proteins of SSTR2 and SSTR5 were expressed in all samples [32,33]. Syro and colleagues sequenced the *SSTR2* and *SSTR5* genes, finding no pathological germline variants in *SSTR2*, but a heterozygous c.143C > A transversion (rs4988483) leading to p.L48M substitution in *SSTR5* was identified in the patient’s blood DNA [34]. 

In a study by Peculis et al., involving 143 cases and 354 controls, the role of SNPs in seven genes (*SSTR2*, *SSTR5*, *DRD2*, *MEN1*, *AIP*, *GNAS*, and *PRKAR1A*) was investigated, focusing on the association with the occurrence of pituitary tumors, phenotype, and clinical symptoms. The study found that rs7131056 in *DRD2* contributes to either faster adenoma growth or less symptomatology, allowing PAs to grow larger before they are detected. However, no significant associations were found for *SSTR2*, while *SSTR5* was implicated in PA development [35].

Pisarek et al. reported an expression pattern of SSTR2B > SSTR2A > SSTR5 in a group of 22 NFPAs, and in another study, Pisarek et al. reported an IHC staining pattern of SSTR 2B = SSTR 3 = SSTR 5 > SSTR 1 = SSTR 2A in prolactinomas. In the three prolactin-secreting adenomas studied, the expression of SSTR2 was stronger than that of SSTR5 [36]. Yu et al. examined SSTR subtypes in various pituitary adenomas via immunohistochemistry, finding SSTR2 staining stronger in TSHoma, acromegaly, and prolactinoma, while SSTR5 was stronger in corticotropinoma and NFPA. Both SSTR2 and SSTR5 were significantly elevated in TSHoma compared to other adenomas.

It has been suggested that the differential efficacy of somatostatin analogs in the postoperative treatment of somatotropinomas and NFPA may be partly due to the differential expression of SSTR1-5 within and between pituitary tumor types. A study using quantitative RT-PCR compared the absolute mRNA copy numbers for all five SSTR isoforms in 23 somatotropinomas and 19 NFPAs [37]. Somatostatin receptor subtype 5 mRNA was highest in somatotropinomas, followed by SSTR2 > SSTR3 >> SSTR1 >>> SSTR4. In contrast, SSTR3 mRNA was highest in NFPA, followed by SSTR2, while SSTR1, SSTR4, and SSTR5 transcripts were only detectable in selected tumors [37]. Another study found that TSH-secreting adenomas express SSTR1, 2A, 3, and 5 mRNA, especially SSTR2A. In addition to the expression of SSTR2 mRNA, the expression of SSTR5 mRNA may also influence tumor shrinkage by somatostatin analogs against TSH-secreting adenomas [38]. High SSTR2 and low SSTR5 immunoreactivity were also found in Cushing’s disease [39]. Tateno et al. reported high expression of SSTR2 mRNA in NFPAs [40]. 

Our study revealed that individuals with the *SSTR2* rs2236750 AG genotype exhibited approximately a 1.6-fold increased risk of developing PAs in both co-dominant and over-dominant genetic models (*p* = 0.043 and *p* = 0.044, respectively). Additionally, significant differences were noted for *SSTR5* rs34037914, showing statistically significant differences between genotypes’ distribution when Micro-PA was compared with the reference group (*p* = 0.022). Also, the TT genotype was associated with a 12-fold higher odds of Micro-PA occurrence (*p* = 0.044). However, these associations did not withstand Bonferroni correction, underscoring the need for further investigation with larger cohorts to deepen our understanding. Notably, while many studies have focused on immunohistochemistry, Peculis et al.’s study found no significant associations between *SSTR2*, *SSTR5* SNPs, and PA.

Around 20% of familial isolated pituitary adenoma (FIPA) cases stem from germline mutations in the aryl hydrocarbon receptor-interacting protein (*AIP*) gene on 11q13. *AIP* mutations mainly lead to somatotrophic and lactotrophic adenomas, often appearing in childhood or young adulthood. *AIP*, initially identified as a co-chaperone, acts as a tumor suppressor gene. While overexpression of wild-type AIP reduces cell proliferation, its silencing stimulates it. AIP interacts with various proteins, including aryl hydrocarbon receptor, Hsp90, and survivin, but the pivotal interaction in pituitary tumorigenesis remains unclear [41]. DNA sequencing of the patient’s normal tissue unveiled a nonsense mutation at nucleotide position 40 within the coding region of the *AIP* gene. Specifically, a cytosine was replaced by a thymidine (c.40 C > T), resulting in the conversion of a Gln codon to a stop codon (Q14X). Notably, all patient tumors exhibited the loss of the wild-type allele of the *AIP* gene [42]. Patients harboring AIP mutations demonstrated a considerably younger age compared to those without AIP mutations [42]. Since the initial identification of *AIP* mutations in FIPA in 2006, over 75 mutations in the AIP gene have been documented [43,44].

The *AIP* mutation occurs in 26% of familial adenomas, based on 4 studies with 341 tumors [16,17,45,46]. Among 163 patients, 19 (11.7%) had germline *AIP* mutations, with an additional 9 patients showing alterations of unclear significance or polymorphisms. *AIP* mutations were found in 20.5% of pediatric patients, 13.3% of sporadic somatotropinoma patients, 11.5% of prolactinoma patients, and 1/16 patients with non-functional pituitary adenoma [47]. The *AIP* mutation occurs much less frequently in sporadic PAs [44,48]. No sporadic GH/PRL-secreting tumors were reported. *AIP* mutations occurred in 5.4%, 4.4%, 3.3%, and 2.4% of PRL-, GH-, and ACTH-secreting tumors, and NFAs, respectively, with about two-thirds being germline mutations. Various types of mutations in the *AIP* gene were observed, indicating random occurrence in sporadic pituitary tumors and emphasizing the role of *AIP* in tumor development [49]. The AIP gene was also identified in FIPA patients from two families in northern Finland [42]. It consists of 6 exons and encodes a 330 amino acid protein containing a peptidylprolyl cis-trans isomerase-like (PPIase-like) domain (amino acids 31–121), a tetratricopeptide repeat (TPR) domain with 3 TPR motifs (aa 179–298), and a c-terminal α-7 helix (Cα-7h) [50]. A study involving 110 Caucasian patients with pituitary adenoma (55 secreting hormones and 55 non-functioning) living in Germany identified AIP mutations in 2.7% of patients. A heterozygous mutation, R16H (c.47G > A), was found in two patients, and a heterozygous G > C alteration in the 3′UTR, 60 bp downstream of the termination codon, was found in one patient. All three patients suffered from a non-functional adenoma. In addition, a silent polymorphism, D172D (c.516C > T), was found in three patients with non-functioning adenoma, two patients with prolactinoma, and one patient with acromegaly [51]. Barlier et al. analyzed 107 patients (prolactinomas (n = 49), non-functioning tumors (n = 29), somatotropinomas (n = 26), ACTH-secreting tumors (n = 2), and TSH-secreting tumors (n = 1)) and found no germline mutations of AIP. In a group of 41 tumor samples from other subjects, a novel AIP mutation (R22X) was found in one sample, in which the corresponding allele was deleted. In a follow-up screening of the patient, a germline AIP mutation, R22X, was detected, suggesting that AIP mutations do not appear to play a major role in sporadic pituitary tumorigenesis in the European population [52]. In our research, we did not find any associations between *AIP* rs267606574 and pituitary adenoma.

Understanding these genetic variations can pave the way for personalized medicine approaches. For example, patients with certain *SSTR2* or *SSTR5* polymorphisms might respond differently to somatostatin analogs, commonly used in PA treatment. Identifying these polymorphisms before treatment could help tailor therapies to individual genetic profiles, potentially improving treatment efficacy and reducing side effects. Our study suggests that genetic screening for *SSTR2* and *SSTR5* polymorphisms could become an integral part of personalized PA management. However, further research with larger cohorts is necessary to validate these findings and explore their clinical applications.

## 5. Conclusions

The binary logistic regression analysis revealed a significant association between the *SSTR2* rs2236750 AG genotype and a 1.6-fold increased likelihood of PA occurrence. Additionally, the *SSTR5* rs34037914 TT genotype was linked to a 12-fold increased odds of Micro-PA occurrence. However, these findings lost significance after Bonferroni correction. This study contributes to understanding the genetic landscape of PAs by identifying potential genetic markers associated with their development. Further research with larger cohorts is essential to validate these findings and deepen our understanding of the molecular mechanisms underlying PA pathogenesis.

## Figures and Tables

**Table 1:** Demographic characteristics of the study.

Characteristics		Group		*p*-Value
		PA Group	Reference Group	
Gender	Males, N (%)	52 (40.6)	86 (31.6)	0.077 *
	Females, N (%)	76 (59.4)	186 (68.4)	
Age, Median (IQR)		54.5 (20)	56.0 (40)	0.821 **
Recurrence: Absent/Present		88/39	NA	-
Invasiveness: Invasive/Noninvasive		81/46	NA	-
Size: Micro/Macro		49/78	NA	-
Activity:			NA	-
Active PA/Nonactive PA		61/66		
Prolactin producing PA		48		
IGF1 producing PA		3		
GF producing PA		6		
ACTH producing PA		4		
Ki-67:			NA	-
<1%		53		
1%		11		
>1%		15		

* Pearson Chi-Square test was used. ** Mann–Whitney U test was used. PA—pituitary adenoma; IQR—interquartile range; *p*-value—significance level (alpha = 0.05).

**Table 2:** Genotype and allele frequencies of single-nucleotide polymorphisms (*SSTR2* rs2236750, *SSTR5* rs34037914, and *AIP* rs267606574) within PA and reference groups.

Gene, SNP	Genotype, Allele	PA Group, *n* (%)	Reference Group, *n* (%)	*p*-Value
*SSTR2* rs2236750	AA	43 (33.6)	118 (43.4)	
	AG	73 (57.0)	126 (46.3)	0.125
	GG	12 (9.4)	28 (10.3)	
	Total	128 (100)	272 (100)	
	Allele			
	A	159 (62.1)	362 (66.5)	0.220
	G	97 (37.9)	182 (33.5)	
*SSTR5* rs34037914	CC	114 (89.1)	238 (87.5)	
	CT	12 (9.4)	33 (12.1)	0.322
	TT	2 (1.6)	1 (0.4)	
	Total	128 (100)	272 (100)	
	Allele			
	C	240 (93.8)	509 (93.6)	0.921
	T	16 (6.2)	35 (6.4)	
*AIP* rs267606574	TAC	128 (100)	272 (100)	
	Total	128 (100)	272 (100)	1.000
	Allele			
	T	128 (33.3)	272 (33.3)	
	A	128 (33.3)	272 (33.3)	1.000
	C	128 (33.3)	272 (33.3)	

PA—pituitary adenoma; *p*-value—significance level (after Bonferroni correction, the results were considered as statistically significant when *p* < 0.025 (0.05/2)).

**Table 3:** Analysis of Hardy–Weinberg equilibrium in the reference group.

Gene and SNP	Allele Frequencies		Genotype Distribution	*p*-Value
*SSTR2* rs2236750	0.67 A	0.33 G	28/126/118	0.505
*SSTR5* rs34037914	0.94 C	0.06 T	1/33/238	0.899
*AIP* rs267606574	NA	NA	0/0/272	NA

SNP—single-nucleotide polymorphism; *p*-value—significance level; NA—not applicable.

**Table 4:** Binary logistic regression analysis within patients with pituitary adenoma and reference group subjects.

Model	Genotype/Allele	OR (95% CI)	*p*-Value	AIC
*SSTR2* rs2236750				
Co-dominant	AG vs. AA GG vs. AA	1.602 (1.015–2.527) 1.170 (0.543–2.522)	0.043 0.688	498.138
Dominant	AG + GG vs. AA	1.524 (0.980–2.371)	0.062	496.848
Recessive	AA vs. GG + AG	0.890 (0.435–1.822)	0.749	500.300
Over-dominant	AG vs. AA + GG	1.550 (1.013–2.373)	0.044	496.298
Adityve	G	1.241 (0.892–1.725)	0.200	498.760
*SSTR5* rs34037914				
Co-dominant	CT vs. CC TT vs. CC	0.781 (0.387–1.576) 4.348 (0.384–49.187)	0.490 0.235	500.343
Dominant	CT + TT vs. CC	0.887 (0.456–1.725)	0.724	500.277
Recessive	TT vs. CC + CT	4.447 (0.393–50.299)	0.228	498.834
Over-dominant	CT vs. CC + TT	0.772 (0.383–1.557)	0.470	499.865
Adityve	T	0.999 (0.550–1.814)	0.997	500.404

PA: pituitary adenoma; OR: odds ratio; CI: confidence interval; *p*-value: significance level (after Bonferroni correction, the results were considered as statistically significant when *p* < 0.025 (0.05/2)); AIC: Akaike information criterion. The most robust genetic model is underlined (selected based on the lowest AIC value).

**Table 5:** Genotype and allele frequencies of *SSTR2* rs2236750 and *SSTR5* rs34037914 within PA and reference groups females.

Gene, SNP	Genotype, Allele	PA Group Females, *n* (%)	Reference Group Females, *n* (%)	*p*-Value
*SSTR2* rs2236750	AA	24 (31.6)	82 (44.1)	0.171
	AG	44 (57.9)	87 (46.8)	
	GG	8 (10.5)	17 (9.1)	
	Total	76 (100)	186 (100)	
	Allele			
	A	92 (60.5)	251 (67.5)	0.129
	G	60 (39.5)	121 (32.5)	
*SSTR5* rs34037914	CC	64 (84.2)	163 (87.6)	
	CT	10 (13.2)	23 (12.4)	0.082
	TT	2 (2.6)	0 (0)	
	Total	76 (100)	186 (100)	
	Allele			
	C	138 (90.8)	349 (93.8)	0.219
	T	14 (9.2)	23 (6.2)	

**Table 6:** Genotype and allele frequencies of single-nucleotide polymorphisms *SSTR2* rs2236750 and *SSTR5* rs34037914 within PA and reference groups: males.

Gene, SNP	Genotype, Allele	PA Group Males, *n* (%)	Reference Group Males, *n* (%)	*p*-Value
*SSTR2* rs2236750	AA	19 (36.5)	36 (41.9)	0.423
	AG	29 (55.8)	39 (45.3)	
	GG	4 (7.7)	11 (12.8)	
	Total	52 (100)	86 (100)	
	Allele			
	A	67 (64.4)	111 (64.5)	0.984
	G	37 (35.6)	61 (35.5)	
*SSTR5* rs34037914	CC	50 (96.2)	75 (87.2)	0.207
	CT	2 (3.8)	10 (11.6)	
	TT	0 (0)	1 (1.2)	
	Total	52 (100)	86 (100)	
	Allele			
	C	102 (98.1)	160 (93)	0.063
	T	2 (1.9)	12 (7)	

**Table 7:** Binary logistic regression analysis within females with pituitary adenoma and reference group males.

Model	Genotype/Allele	OR (95% CI)	*p*-Value	AIC
*SSTR2* rs2236750				
Co-dominant	AG vs. AA GG vs. AA	1.722 (0.962–3.083) 1.600 (0.615–4.164)	0.067 0.335	315.898
Dominant	AG + GG vs. AA	1.702 (0.968–2.993)	0.065	313.923
Recessive	AA vs. GG + AG	1.165 (0.480–2.828)	0.735	317.324
Over-dominant	AG vs. AA + GG	1.560 (0.910–2.675)	0.106	314.796
Adityve	G	1.402 (0.923–2.131)	0.114	314.927
*SSTR5* rs34037914				
Co-dominant	CT vs. CC TT vs. CC	1.117 (0.502–2.486) -	0.786 0.999	314.452
Dominant	CT + TT vs. CC	1.345 (0.630–2.869)	0.444	316.864
Recessive	TT vs. CC + CT	-	0.999	312.525
Over-dominant	CT vs. CC + TT	1.088 (0.490–2.420)	0.835	317.394
Adityve	T	1.526 (0.771–3.023)	0.225	316.008

PA: pituitary adenoma; OR: odds ratio; CI: confidence interval; *p*-value: significance level (after Bonferroni correction, the results were considered as statistically significant when *p* < 0.025 (0.05/2)); AIC: Akaike information criterion. The most robust genetic model is underlined (selected based on the lowest AIC value).

**Table 8:** Binary logistic regression analysis within males with pituitary adenoma and reference group males.

Model	Genotype/Allele	OR (95% CI)	*p*-Value	AIC
*SSTR2* rs2236750				
Co-dominant	AG vs. AA GG vs. AA	1.449 (0.690–3.044) 0.736 (0.202–2.685)	0.327 0.643	184.776
Dominant	AG + GG vs. AA	1.305 (0.635–2.682)	0.468	183.976
Recessive	AA vs. GG + AG	0.591 (0.176–1.990)	0.396	183.743
Over-dominant	AG vs. AA + GG	1.544 (0.770–3.096)	0.221	182.996
Adityve	G	1.039 (0.605–1.786)	0.889	184.486
*SSTR5* rs34037914				
Co-dominant	CT vs. CC TT vs. CC	0.295 (0.062–1.408) -	0.126 1	182.751
Dominant	CT + TT vs. CC	0.271 (0.058–1.278)	0.099	181.054
Recessive	TT vs. CC + CT	-	1	183.649
Over-dominant	CT vs. CC + TT	0.298 (0.062–1.422)	0.129	181.656
Adityve	T	0.279 (0.062–1.267)	0.098	180.844

PA: pituitary adenoma; OR: odds ratio; CI: confidence interval; *p-*value: significance level (after Bonferroni correction, the results were considered as statistically significant when *p* < 0.025 (0.05/2)); AIC: Akaike information criterion. The most robust genetic model is underlined (selected based on the lowest AIC value).

**Table 9:** Genotype and allele frequencies of single-nucleotide polymorphisms *SSTR2* rs2236750 and *SSTR5* rs34037914 with Micro-PA or Macro-PA and reference groups.

Gene, SNP	Genotype, Allele	Reference Group, *n* (%)	Micro-PA Group, *n* (%)	Macro-PA Group, *n* (%)	*p*-Value
*SSTR2* rs2236750	AA	118 (43.4)	18 (38.3)	25 (30.9)	0.423 *
	AG	126 (46.3)	25 (53.2)	48 (59.3)	0.103 **
	GG	28 (10.3)	4 (8.5)	8 (9.9)	0.691 ***
	Total	272 (100)	47 (100)	81 (100)	
	Allele				0.754 *
	A	362 (66.5)	61 (64.9)	98 (60.5)	0.156 **
	G	182 (33.5)	33 (35.1)	64 (39.5)	0.484 ***
*SSTR5* rs34037914	CC	238 (87.5)	42 (89.4)	72 (88.9)	0.022 *
	CT	33 (12.1)	3 (6.4)	9 (11.1)	0.833 **
	TT	1 (0.4)	2 (4.3)	0 (0.0)	0.125 ***
	Total	272 (100)	47 (100)	81 (100)	
	Allele				0.715 *
	C	509 (93.6)	87 (92.6)	153 (94.4)	0.685 **
	T	35 (6.4)	7 (7.4)	9 (5.6)	0.547 ***

* Micro-PA group vs. reference group. ** Macro-PA group vs. reference group. *** Micro-PA vs. Macro-PA.

**Table 10:** Binary logistic regression analysis within Micro- or Macro-PA and reference group subjects.

Model	Genotype/Allele	OR (95% CI)	*p*-Value	AIC
Micro-PA				
*SSTR2* rs2236750				
Co-dominant	AG vs. AA GG vs. AA	1.301 (0.675–2.506) 0.937 (0.294–2.985)	0.432 0.912	269.952
Dominant	AG + GG vs. AA	1.234 (0.654–2.330)	0.516	268.295
Recessive	AA vs. GG + AG	0.811 (0.271–2.427)	0.708	268.575
Over-dominant	AG vs. AA + GG	1.317 (0708–2.449)	0.385	267.965
Adityve	G	1.081 (0.673–1.737)	0.747	268.618
*SSTR5* rs34037914				
Co-dominant	CT vs. CC TT vs. CC	0.515 (0.151–1.756) 11.333 (1.005–127.806)	0.289 0.050	265.188
Dominant	CT + TT vs. CC	0.833 (0.308–2.253)	0.719	268.588
Recessive	TT vs. CC + CT	12.044 (1.070–135.599)	0.044	264.500
Over-dominant	CT vs. CC + TT	0.494 (0.145–1.681)	0.259	267.217
Adityve	T	1.156 (0.515–2.593)	0.726	268.602
Macro-PA				
*SSTR2* rs2236750				
Co-dominant	AG vs. AA GG vs. AA	1.798 (1.043–3.100) 1.349 (0.550–3.305)	0.035 0.513	379.660
Dominant	AG + GG vs. AA	1.716 (1.011–2.913)	0.045	378.113
Recessive	AA vs. GG + AG	0.955 (0.417–2.186)	0.913	282.257
Over-dominant	AG vs. AA + GG	1.685 (1.019–2.788)	0.042	378.074
Adityve	G	1.333 (0.911–0.952)	0.139	380.090
*SSTR5* rs34037914				
Co-dominant	CT vs. CC TT vs. CC	0.902 (0.412–1.972) -	0.795 -	383.679
Dominant	CT + TT vs. CC	0.875 (0.401–1.910)	0.737	382.155
Recessive	TT vs. CC + CT	-	-	-
Over-dominant	CT vs. CC + TT	0.905 (0.414–1.980)	0.803	382.206
Adityve	T	0.853 (0.399–1.826)	0.682	382.097

PA: pituitary adenoma; OR: odds ratio; CI: confidence interval; *p*-value: significance level (after Bonferroni correction, the results were considered as statistically significant when *p* < 0.025 (0.05/2)); AIC: Akaike information criterion. The most robust genetic model is underlined (selected based on the lowest AIC value).

**Table 11:** SSTR2 serum levels in patients with pituitary adenoma and in reference groups by gender.

Gender	Serum Level (pg/mL)		*p*-Value
	PA Group Mean (Std. Deviation)	Reference Group Mean (Std. Deviation)	
SSTR2			
Male	9643.05 (3797.57)	8004.66 (3660.49)	0.122
Female	9159.36 (4819.88)	9501.08 (3581.27)	0.778

**Table 12:** SSTR5 serum levels in patients with pituitary adenoma and in reference groups by gender.

Gender	Serum Level (pg/mL)		*p*-Value
	PA Group Median (IQR)	Reference Group Median (IQR)	
SSTR5			
Male	190.75 (209.98)	247.74 (201.46)	0.490
Female	197.56 (396.82)	255.09 (746.07)	0.675

**Table 13:** AIP serum levels in patients with pituitary adenoma and in reference groups by gender.

Gender	Serum Level (ng/mL)		*p*-Value
	PA Group Mean (Std. Deviation)	Reference Group Mean (Std. Deviation)	
AIP			
Male	0.242 (0.055)	0.303 (0.130)	0.149
Female	0.250 (0.121) ng/mL	0.246 (0.056)	0.943

**Table 14:** Ki-67 labeling index considering invasiveness of pituitary adenoma.

Invasiveness	Ki-67 LI			*p*-Value
	<1%	1%	>1%	
Noninvasive PA n = 22 (27.8%)	16 (72.7%)	2 (9.1%)	4 (18.2%)	0.717
Invasive PA n = 57 (72.2%)	37 (64.9%)	9 (15.8%)	11 (19.3%)	

**Table 15:** Ki-67 labeling index considering recurrence of pituitary adenoma.

Recurrence	Ki-67 LI			*p*-Value
	<1%	1%	>1%	
Absent n = 56 (70.9%)	38 (67.9%)	7 (12.5%)	11 (19.6%)	0.843
Present n = 23 (29.1%)	15 (62.2%)	4 (17.4%)	4 (17.4%)	

**Table 16:** Ki-67 labeling index considering the activity of pituitary adenoma.

Activity	Ki-67 LI			*p*-Value
	<1%	1%	>1%	
Active PA n = 44 (55.7%)	31 (70.5%)	4 (9.1%)	9 (20.5%)	0.378
Nonactive PA n = 35 (44.3%)	122 (62.9%)	7 (20.0%)	6 (17.1%)	

**Table 17:** Ki-67 labeling index considering the size of pituitary adenoma.

Size	Ki-67 LI			*p*-Value
	<1%	1%	>1%	
Micro-PA n = 31 (39.2%)	23 (74.2%)	4 (12.9%)	4 (19.9%)	0.492
Macro-PA n = 48 (60.8%)	30 (62.2%)	7 (14.6%)	11 (22.9%)	

**Table 18:** Ki-67 labeling index associations with *SSTR2* rs2236750, *SSTR5* rs34037914, and *AIP* rs267606574.

Gene, SNP	Genotype/Allele	Ki-67 LI			*p*-Value
		<1%	1%	>1%	
*SSTR2* rs2236750	Genotype	20 (37.7)	5 (45.5)	4 (26.7)	0.636
	AA	27 (50.9)	4 (36.4)	10 (66.7)	
	AG	6 (11.3)	2 (18.2)	1 (6.7)	
	GG	53 (100)	11 (100)	15 (100)	
	Total				
	Allele				
	A	67 (63.2)	14 (62.6)	18 (60.0)	
	G	39 (36.8)	8 (36.4)	12 (40.0)	0.945
*SSTR5* rs34037914	Genotype	47 (88.7)	9 (81.8)	15 (100)	0.087
	CC	6 (11.3)	1 (9.1)	0 (0.0)	
	CT	0 (0.0)	1 (9.1)	0 (0.0)	
	TT	53 (100)	11 (100)	15 (100)	
	Total				
	Allele				
	C	100 (93.6)	19 (86.4)	30 (100)	
	T	6 (6.4)	3 (13.6)	0 (0)	0.275

## Data Availability

All data could be seen in this manuscript.
